# Students’ attitude and sleep pattern during school closure following COVID-19 pandemic quarantine: a web-based survey in south of Iran

**DOI:** 10.1186/s12199-021-00950-4

**Published:** 2021-03-10

**Authors:** Keivan Ranjbar, Hamidreza Hosseinpour, Reza Shahriarirad, Haleh Ghaem, Khoubyar Jafari, Tayebeh Rahimi, Alireza Mirahmadizadeh, Parisa Hosseinpour

**Affiliations:** 1 Thoracic and Vascular Surgery Research Center, Shiraz University of Medical Sciences, Shiraz, Iran; 2 Student Research Committee, Shiraz University of Medical Sciences, Shiraz, Iran; 3 Department of Surgery, Shiraz Laparoscopic Research Center, Shiraz University of Medical Sciences, Shiraz, Iran; 4 Non-communicable Diseases Research Center, Shiraz University of Medical Sciences, Shiraz, Iran; 5 Teenagers and Youth Health Department of Shiraz University of Medical Sciences, Shiraz, Iran; 6 Fars Education Counselling Office, Shiraz, Iran; 7 School of Medicine, Islamic Azad University, Kazeroun branch, Kazeroun, Iran

**Keywords:** School, Education, Pandemic, COVID-19, Activities, Sleep, Attitude

## Abstract

**Background:**

School closure is one of the main policies of global health care strategies performed worldwide. Despite all benefits, there might be some threats for younger groups spending their time in quarantine. This study aims to determine the impacts of lockdown and school closure on children’s major lifestyle aspects, especially their leisure and sleep pattern during the COVID-19 pandemic.

**Methods:**

For the purpose of this study, an online questionnaire was distributed from 14th to 31st of March 2020 among the schools and students from the first grade to the 12th grade (before university) in Fars province, southern Iran. The questionnaire consisted of five sections which included data regarding the students’ general information, activity priorities, adherence to quarantine, attitude toward school closure, and sleep patterns.

**Results:**

In our study, 20,697 filled questionnaires were received from the participants with an average age of 13.76 years; 29.7% of them were male, 80.6% were from urban areas, and 83.3% were from public schools. The overall first preference of students during school closure was mobile and computer games (30.1%), followed by studying (26.6%) and watching television (13.8%). Our results demonstrated that the majority of students adhered to social distancing and there was also a significant correlation among education levels and desire for schools to be closed till the end of the semester (*P* = 0.015). Also, regarding sleep patterns, the majority (53.5%) had above 12 h of sleep throughout the day.

**Conclusion:**

It seems that lockdown following COVID-19 pandemic has changed various aspects of the students’ lifestyle remarkably, especially by increasing screen time and even sleep duration and pattern. We believe that certain strategies should be implemented by the Health and Educational Ministry to control not only the visible side effects of the quarantine period, but also the collateral consequences on their psychological and mental health.

**Supplementary Information:**

The online version contains supplementary material available at 10.1186/s12199-021-00950-4.

## Background

As an officially accepted pandemic, the novel coronavirus disease 2019 (COVID-19) has had various financial and life effects on the world’s population until now [1]. However, these threatening psychosocial effects should not be overlooked, as mental health is a crucial part of public health in which factors such as lockdown are the primary key factor of various psychopathological problems [2, 3].

Children are one of the prominent victims of the performed restricted quarantine strategies’ effects, becoming vulnerable to various mental health problems [4]. These extensive changes, such as school closure and continuous contact with family, have made substantial effects on their daily routines, including leisure time activities, sleep, and even social communications [5]. Altering sleep pattern and less physical activity affect the children’s physical and mental health itself, which may subsequently result in consequences such as gaining weight, home violence, post-traumatic psychological stress, decreased social contacts, and increased screen time [6, 7].

Therefore, the first and one of the most crucial steps of the management of these collateral consequences is recognizing the changes in children’s daily routine variables, with the intention of assisting policymakers in following the children’s potential behavioral patterns and, ultimately, taking necessary measures [8]. Although several studies have suggested various strategies to deal with the psychological effects of the lockdown, the need for a universal, coordinated, and comprehensive strategy, especially during the COVID-19 pandemic, is still indispensable [9].

In this survey, we aimed to find out how children, especially during school closure caused by the COVID-19 pandemic, spend their time during the lockdown and also to evaluate their sleep patterns and social communications in detail. We also discuss some strategies to handle the majority of behavioral and psychological consequences.

## Methods and materials

### Study area

The target population of our study consisted of all students in Fars province, which is the fourth largest province in Iran with a population of 4,851,274 individuals (in 2016), and Shiraz, the capital of this province, with a population of 1,869,001(the fifth populated city in Iran). Based on statistics reports till 2020, the province consists of 9696 schools, 41,340 classrooms, and 895,042 students, including 440,708 female and 454,334 male students [10].

### Sample size

Sample size estimation was performed based on a study by Erfani et al. [11] and by considering a confidence level of 95%, with a *d* (margin of error) of 0.01, minimum proportion of good attitude and sleep equaling to 30%, and 8067 participants calculated, and based on the variations in the living locations and type of schools (private vs public) and applying a design effect of 2.5, reaching a final sample size of approximately 20,200 participants.

### Questionnaire preparation

For the purpose of this study, we designed a questionnaire which was developed for the evaluation of the students’ activities during quarantine and their priorities. The questionnaire consisted of five sections: (I) details of the participant’s age, gender, living location, type of school, educational level, and grade point average (GPA); (II) a list of nine activities which the students had to arrange based on their priority of activities they engage in their leisure time during school closure; (III) four questions regarding the students’ adherence to quarantine; (IV) nine statements regarding the students’ attitude toward school closure which was scored on a basis of a five-point Likert scale including completely disagree, disagree, no opinion, agree, and completely agree; (V) two questions regarding the usual and most frequent time they went to bed at night and the time they wake up in the morning during school closure.

The questionnaire was subsequently pilot tested. Before the final survey was completed, changes were made as required to enable a better understanding of the questions by the participants, and the arrangement of the questions was checked to ensure its efficiency. The final version of the questionnaire took approximately 3 minutes to be filled out.

### Data collection

The questionnaire was carried out from 14th to 31st of March 2020. For data collection and the distribution of our questionnaire, the Vice-Chancellor for Health Affairs of the Ministry of Education, who is responsible for health and hygiene and performs supervision and necessary examinations in school districts, handed out the questionnaire to each schools’ health coach, which subsequently distributed the questionnaire through the school’s specific online family network system established for monitoring the students during the COVID-19 pandemic school closure period. The cover page of the survey contained a brief overview of the purposes, voluntary nature of participation, procedures, and statements of confidentiality and anonymity. The participants could view the questions basically by entering the provided link and answering the questions. The inclusion criteria of our study consisted of Fars province, southern Iran students from the first grade to the 12th grade (before university). For students who were younger or did not understand the content of the survey, we asked their parents or guardian to assist in filling out the questionnaire. Also, IP filtering was used to guard against duplicate responses and participants could withdraw anytime they wished during the survey.

### Statistical analysis

All the statistical analyses were performed by the statistical package for social sciences (SPSS Inc., Chicago, IL, USA) version 26.0. Data are presented as mean ± standard deviation (SD) or median and interquartile range (IQR) as appropriate. Parametric data with normal distribution were used to compare between groups using an independent *t* test while those without normal distribution were compared using Mann Whitney *U* test.

## Results

### Demographic features

In our study, 20697 filled questionnaires were received from the participants with an average age of 13.76 ± 2.50 which included 6139 (29.7%) male and 14558 (70.3%) females. Among the participants, 16672 (80.6%) were from urban areas and 4025 (19.4%) from rural areas. The demographic and school features of the students in our study are demonstrated in Table 1.

**Table 1:** Demographic features of students in Fars province in our study

Variables	Groups	Frequency (%)
**Age group**		
	6–9	1594 (7.7)
	10–12	3055 (14.8)
	13–15	11379 (55)
	≥ 16	4669 (22.6)
**Gender**		
	Male	6139 (29.7)
	Female	14558 (70.3)
**School type**		
	Public	17238 (83.3)
	Private	3459 (16.7)
**Living location**		
	Urban	16672 (80.6)
	Rural	4025 (19.4)
**Residence**		
	Shiraz, Capital of Fars	11305 (54.6)
	Others	9392 (45.4)
**Education level**		
	1–3	1752 (8.6)
	4–6	2330 (11.3)
	7–9	11798 (57)
	10–12	4387 (21.2)
**GPA (average from a total score of 20)**		
	≤ 15	944 (4.6)
	15–18	3956 (19.1)
	>18	12738 (86.2)

### Socializing of students

In our study, four questions were asked regarding the students’ adherence to quarantine; the results are demonstrated in Table 2. Our results revealed that the majority of students were adherent to social distancing. Additional file 1 shows these results based on the variables in our study.

**Table 2:** Students’ adherence to quarantine during the COVID-19 pandemic school closures

Variable	Frequency during the past two weeks		
	0	1–2	> 2
Visiting friends or family	10698 (51.7%)	6688 (32.3%)	3311 (16%)
Being visited by friends or family	11639 (56.2%)	6123 (29.6%)	2935 (14.2%)
Shopping or visiting shopping centers	12156 (58.7%)	5735 (27.7%)	2806 (13.6%)
For entertainment and outside activities	14186 (68.5%)	4724 (22.8%)	1787 (8.6%)

Public school children had significantly lower rates of encountering outdoor activities during the quarantine and school closures. Based on the living area, students from rural areas had higher rates of visiting family and friends, also being visited by family and friends compared with those from the cities (*P* < 0.001); however, students from cities had a higher number in shopping and visiting shopping centers compared to rural students (*P* < 0.001) (Additional file 1).

Furthermore, students in lower age groups and also lower educational level stayed at home more often compared to higher age and higher education level ones (*P* < 0.001 in both categories). Based on the GPA of the students, a higher GPA was related to fewer episodes of visiting family and friends, being visited by them, visiting shopping centers, and also outdoor activities (*P* < 0.001).

Based on gender, female students engaged more in outdoor activities, but less frequently in visiting shopping centers and visiting family and friends (*P* < 0.001).

### Students’ attitude toward school closure

A series of nine questions were asked from the students regarding their attitude towards school closure, in which the results are demonstrated in Table 3. Based on chi-square analysis regarding demographic variables of our study and the answers regarding the students’ attitudes, gender had a significant correlation with feeling upset during school closure (question 5) (*P* = 0.021), with female students disagreeing more compared to male students (17.2% vs. 15.9%). Furthermore, the type of school had a significant correlation with students missing their teachers (question 4) (*P* = 0.023), with public school students responding as agree and completely agree more frequently than private school students (80.6% vs.77.9%). There was also a significant correlation among education levels and desire for schools to be closed till the end of the semester (question 9) (*P* = 0.015), with the highest agreement rate in education levels 1 to 3, while the highest disagreement rates in level 10 to 12. There was no significant correlation among the students’ living area and grades with the received answers regarding their attitude toward school closure.

**Table 3:** Frequency of answers regarding the students’ attitudes toward school closure

No.	Question	Answer (%)				
		Completely agree	Agree	No opinion	Disagree	Completely disagree
1	Till the official opening, I will not exit the house	13355 (64.5)	4171 (20.2)	1722 (8.3)	839 (4.1)	610 (2.9)
2	I miss going to school	11466 (55.4)	5199 (25.1)	1965 (9.5)	864 (4.2)	1203 (5.8)
3	I miss seeing my friends	14221 (68.7)	3977 (19.2)	1148 (5.5)	482 (2.3)	869 (4.2)
4	I miss seeing my teachers	11960 (57.8)	4470 (21.6)	1986 (9.6)	751 (3.6)	1530 (7.4)
5	The longer the quarantine and school closure lasts, the more upset I become	8562 (41.4)	4511 (21.8)	4150 (20.1)	1691 (8.2)	1783 (8.6)
6	If I could exit the house, I prefer schools to be closed	3663 (17.7)	2530 (12.2)	4618 (22.3)	4877 (23.6)	5009 (24.2)
7	I feel that I’m behind on my studies	6502 (31.4)	5544 (26.8)	2981 (14.4)	3010 (14.5)	2660 (12.9)
8	I feel confused since I don’t know what to do	6237 (30.1)	5461 (26.4)	3341 (16.1)	2757 (13.3)	2901 (14)
9	Schools should be closed till the end of the semester no matter what	7529 (36.4)	3479 (16.8)	5214 (25.2)	2015 (9.7)	2460 (11.9)

### Sleep hours

**Fig. 1:**
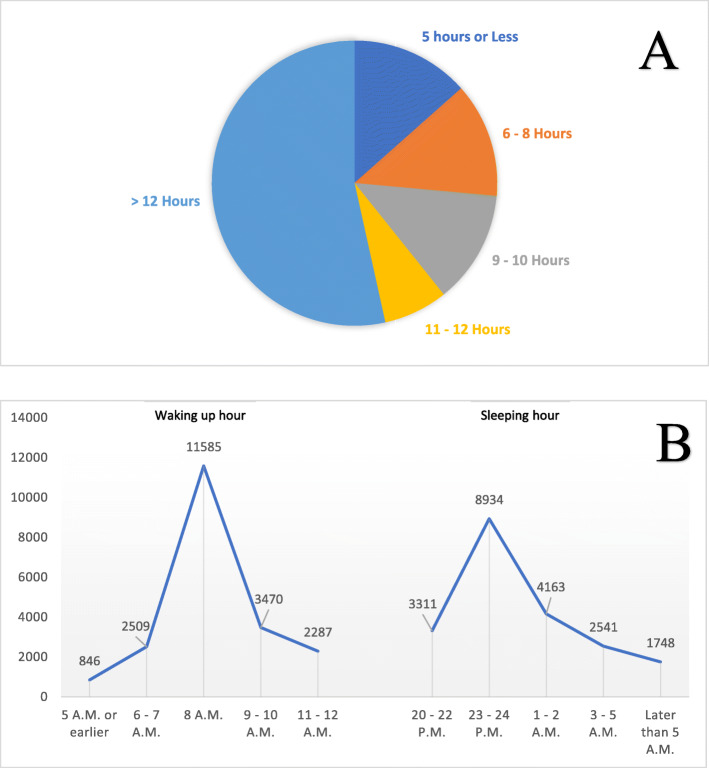
(**a**) Frequency of students in each night-time sleep hour during school closure. (**b**) Time of waking up and sleeping among students during school closure due to the COVID-19 pandemic

Based on our results, 2782 (13.4%) students had 5 or fewer hours of sleep, 2689 (13%) had 6 to 8 h, 2655 (12.8%) had 9 to 10 h, 1506 (7.3%) had 11 to 12 h, and 11065 (53.5%) had above 12 h of sleep throughout the day. As demonstrated in Fig. 1, the majority (8934: 43.2%) of students went to bed between 23 and 24 P.M. while the majority (11585: 56%) woke up at 8 A.M.

### Attitude

The attitude and activities of the students were categorized into nine subgroups which were sorted based on their preference. Table 4 demonstrates the first preference of the students based on the variables in our study.

**Fig. 2:**
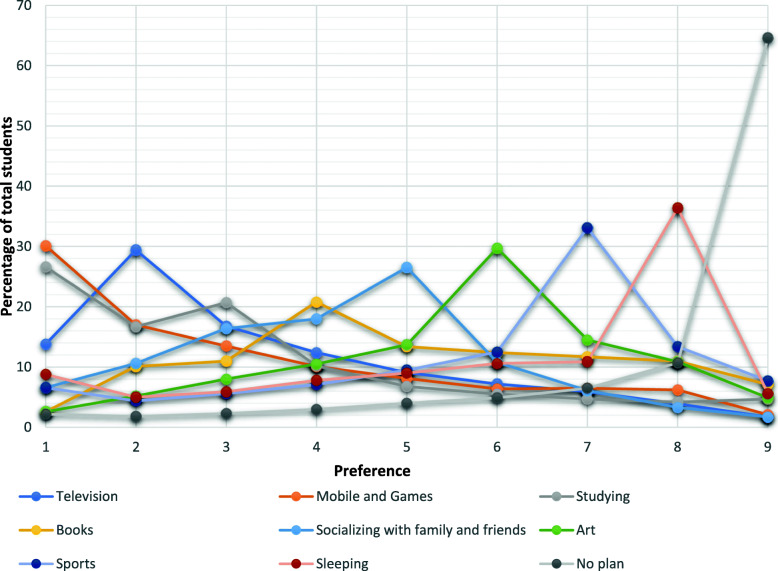
The overall percentage of activity preference among students during the school closure

As demonstrated in Table 4, the overall first preference of students during school closure was mobile and computer games (30.1%), followed by studying (26.6%) and watching television (13.8%). The overall trend of preferences is demonstrated in Fig. 2, with students reporting their first to ninth preference as playing mobile and computer games, watching television, studying, reading books, socializing with family and friends, doing art activities and sports, sleeping, and having no plan, respectively.

**Table 4:** The first preference of students during school closure due to the COVID-19 pandemic

Variable	Activity (%)								
	Television	Mobile and computer games	Studying	Books	Socializing with family and friends	Art	Sports	Sleeping	No plan
**Total**	2851 (13.8)	6225 (30.1)	5513 (26.6)	535 (2.6)	1369 (6.6)	546 (2.6)	1373 (6.6)	1820 (6.6)	1820 (8.8)
**Gender**									
Male	974 (15.9)	2165 (35.3)	1572 (25.6)	129 (2.1)	341 (5.6)	91 (1.5)	370 (6)	386 (6.3)	111 (1.8)
Female	1877 (12.9)	4060 (27.9)	3941 (27.1)	406 (2.8)	1028 (7.1)	455 (3.1)	1003 (6.9)	1434 (9.9)	354 (2.4)
**Age group**									
6–9	499 (31.3)	446 (28)	361 (22.6)	19 (1.2)	68 (4.3)	32 (2)	74 (4.6)	87 (5.5)	8 (0.5)
10–12	592 (19.4)	793 (26)	828 (27.1)	83 (2.7)	183 (6)	70 (2.3)	250 (8.2)	206 (6.7)	50 (1.6)
13–15	1421 (12.5)	3344 (29.4)	3157 (27.7)	300 (2.6)	775 (6.8)	289 (2.5)	857 (7.5)	980 (8.6)	256 (2.2)
≥ 16	339 (7.3)	1642 (35.2)	1167 (25)	133 (2.8)	343 (7.3)	155 (3.3)	192 (4.1)	547 (11.7)	151 (3.2)
**Type of school**									
Public	2340 (13.6)	5166 (30)	4625 (26.8)	442 (2.6)	1130 (6.6)	461 (2.7)	1153 (6.7)	1525 (8.8)	396 (2.3)
Private	511 (14.8)	1059 (30.6)	888 (25.7)	93 (2.7)	239 (6.9)	85 (2.5)	220 (6.4)	295 (8.5)	69 (2)
**Living location**									
Rural	549 (13.6)	1115 (27.7)	1230 (30.6)	74 (1.8)	266 (6.6)	76 (1.9)	363 (9)	274 (6.8)	78 (1.9)
Urban	2302 (13.8)	5110 (30.7)	4283 (25.7)	461 (2.8)	1103 (6.6)	470 (2.8)	1010 (6.1)	1546 (9.3)	387 (2.3)
**Education level**									
1–3	517 (29.5)	501 (28.6)	409 (23.3)	22 (1.3)	81 (4.6)	37 (2.1)	86 (4.9)	88 (5)	11 (0.6)
4–6	454 (19.5)	656 (28.2)	598 (25.7)	63 (2.7)	138 (5.9)	55 (2.4)	183 (7.9)	145 (6.2)	38 (1.6)
7–9	1502 (12.7)	3422 (29)	3304 (28)	307 (2.6)	795 (6.7)	296 (2.5)	917 (7.8)	1000 (8.5)	255 (2.2)
10–12	314 (7.2)	1526 (34.8)	1086 (24.8)	134 (3.1)	331 (7.5)	145 (3.3)	157 (3.6)	544 (12.4)	150 (3.4)
**GPA (average from a total score of 20)**									
≤ 15	128 (13.6)	375 (39.7)	195 (20.7)	26 (2.8)	59 (6.3)	16 (1.7)	55 (5.8)	63 (6.7)	27 (2.9)
15–18	463 (11.7)	1424 (36)	889 (22.5)	105 (2.7)	253 (6.4)	105 (2.7)	238 (6)	370 (9.4)	109 (2.8)
> 18	1943 (15.3)	3474 (27.3)	3632 (28.5)	317 (2.5)	839 (6.6)	326 (2.6)	882 (6.9)	1091 (8.6)	234 (1.8)

Furthermore, the students’ gender followed the overall trend pattern of preferences (Additional file 2). However, the trend of preferences varied based on age groups, with students between 6 and 9 years of age preferring watching television, while 10- to 12-year-old students preferred studying, and above 12-year-old ones preferred mobile and computer games. Based on the other preferences trend, all age groups reported reading books, socializing with family and friends, doing art activities, sports, sleeping throughout the day, and having no plan as the fourth to ninth preference, respectively, except the 16 and above year old group which preferred socializing with family and friends as both the fourth and fifth preference. As our data indicate, for 6- to 9-year-old students, the first and second preference was watching television while third was studying. This trend for 10- to 12-year-old students differed; for them, studying was the first and third preferences, while watching television was the second one. For 13- to 15-year-old ones, the first preference was playing mobile and computer games, while the second one was playing television, and the third preference was studying. For 16 and above, the first was mobile and computer games, while the second and third preference was watching television and fourth and fifth, as mentioned before, was socializing with family and friends (as provided in Additional file 3).

The trend of the preference of activity based on educational level was similar to that based on age groups, with a small difference in the education levels 4 to 6 which preferred mobile and computer games (28.2%) compared to studying (25.7%) (as provided in Additional file 4).

Based on the living area, students from rural areas preferred studying, while those from the cities preferred mobile and computer games as their primary activity. The remaining preference trend was similar to the overall pattern (as provided in Additional file 5).

The trend of activity preference was similar among public and private school students and they followed the overall pattern trend (as provided in Additional file 6).

Regarding the GPA of the students, the preference activity followed the overall trend except in the GPA of above 18 groups in which their first preference was studying (28.5%), followed by mobile and computer games (27.3%), while in lower than 18 GPA students, their first preference was mobile and video games (36% for between 15 and 18 and 39.7% for lower than 15 GPA). Additional file 7 demonstrates the activity preference trends based on GPA.

Among the variables, only the type of school did not have a significant correlation with the preference of activities (*P* > 0.05).

## Discussion

### Study importance

As an effective intervention to control the spread of the COVID-19, school closure is one of the main parts of the quarantine strategies, which has been performed worldwide [12]. Children also have a significant role in transmitting the viruses, especially during pandemics [13]; on the other hand, staying full-time at home has destructive and irrecoverable consequences on their physical and, more importantly, mental health [14, 15]. In this web-based survey, we evaluated the students’ lifestyles, socialization, sleep patterns during the lockdown, and how they switch their leisure and activities and even their attitude toward school closure.

### Activities

According to our study, the overall favorite activity during school closure was mobile and computer games (30.1%), followed by studying (26.6%) and watching television (13.8%). Although socializing with family and friends was also one of their options to choose from in the questionnaire, it seems that students prefer spending their free time alone. The first consequence of these three leading leisure time activities would be weight gain; Pietrobelli et al. claimed that obesity is an apparent negative change of lockdown period [16]. The following collateral side effect of this lifestyle would be the increased chance of depression and aggressive behaviors; as such, there is a strong association between these two psychological aliments and increased screen time [17, 18].

It is also revealed that the trend of leisure preferences of students varied significantly based on their age. As mentioned in the results, this trend switched from watching television in lower aged children (6 to 9 years old) to mobile and computer games in those older than 12 years. Meanwhile, only children aged 10 to 12 years old preferred studying as the primary leisure during their free time. All these results demonstrate how COVID-19 and lockdown, despite the presence of digital educational methods, cause a break in the students’ education process; consequently, the educational gap is identified as a risk factor for loss of academic achievements, as noted by a recent study by Lanker et al. [19].

### Attitude

Notably, students living in rural areas had a significant higher rate of visiting family and friends, but urban students notably had preferred shopping and visiting shopping centers as the outdoor activity. Even though it has clearly resulted from the difference of rural and urban lifestyle and strength of family ties, this difference may also exist due to their level of awareness toward COVID-19; therefore, our study revealed that students with higher GPA were more adherent to quarantine and had significantly fewer family meetings and outdoor activities. Then again, the more adhere to quarantine restrictions, the higher they are exposed to mental and psychological problems; a similar study conducted by Zhou et al. showed that the prevalence of COVID-19’s psychological consequences negatively correlated with the level of alertness about the pandemic [20].

There was also a significant difference between private and public school students in missing their teacher; those studying in public schools had missed their teacher more frequently. This is in contrast with the hypothesis that students who study in private schools may receive more attention and kindness from their educators than their families due to their socioeconomic status [21], as we see private school’s stressful atmosphere put more pressure on students that might make them more reluctant to attend their school. It may also be attributed to their level of comfort and leisure tools provided in their home [22].

### Sleep patterns

The majority of respondents (53.5%) reported that they had more than 12 h of sleep per 24-h day. It seems that besides lower physical activity, sleep pattern changes, and higher sleep hours are recorded throughout the day. According to previous studies, increasing sleep duration is expected in both children and adults during lockdown [23–25]. However, our results differ slightly from those of Cellini et al. in the association of sleep pattern and screen time of children, as they found no correlation between these two variables [26]; however, in our experience, we noticed that children had more sleep duration relative to increased digital media use. On the other hand, we noticed the correlation between increased sleep time and decreased rate of students’ tendency toward art and sports. As we see, sport and art activities were by far among the last preferences of children during the lockdown.

### Comparison to non-pandemic state

Children and adolescents’ mental health has always been important for health care providers in Iran, even in the non-pandemic state, as various studies have evaluated the behavioral status using in-person interviews [13, 27, 28]. According to studies, there is a strong relationship among Iranian sleep patterns, physical activities, and social communications of families and children with psychological functioning and future mental and physical diseases in young people [29, 30]. For instance, Javadieh et al. demonstrated that sleep hours during school days significantly differ from holidays and Fridays [31]. Even though these studies recommend careful supervision on children’s psychological and behavioral aspects even in the absence of pandemic state, we hypothesized that social distancing, especially during school closure, would simulate situations such as holidays and consequently result in undesirable effects of the student’s attitude and sleep patterns.

### Current standing and recommendations

Even though we have insufficient knowledge regarding the long-term mental health effects of the COVID-19 outbreak, it can be assumed that changes in mental health principles, including social communication, leisure activities, and sleep patterns, would result in considerable psychological and behavioral consequences. These effects may also cause collateral consequences such as increased domestic violence rates, educational loss, and depression [32–34]; finally, this vicious cycle would cause irretrievable effects on society.

In summary, the main strategies of educators and mental health care providers should focus on shifting the priorities of students from more sleep and less art and sport to activities which are a combination of their desires and what we believe is good for them; for instance, we can make computer games which include their educational materials and lessons, or develop and merge physical activities with computer games. We could also regulate their sleep pattern by involving sleep guidance in computer games and control their going to bed and waking time. As they get more used to these kinds of activities, they could even gain extra points in their digital games by studying more lessons, and subsequently engage them in more productive activities.

It is worth mentioning that our study was conducted during a specific condition and time period. The situation that was imposed by the COVID-19 pandemic made the Iran government to establish rules and policies to reduce the virus spreading including the closure of all elementary, middle, and high schools from the 29th of February 2020 all over the country, as well as our province (Fars) [35, 36]. In the first weeks, no virtual education was implemented; however, in April 2020, various mobile and computer software were introduced to continue the school year. By this time, they passed the school closure as a law along with the closure of high-risk businesses and tourist attractions [37, 38]. Meanwhile, all intercity road trips were forbidden and non-residents were not allowed to enter the cities; however, public transportation were still available [39]; nevertheless, social distancing and quarantine, although highly recommended, was not enforced. During this period, Iran’s education ministry determined to implement social distancing rules and decided that the schools will remain closed [40]. Also, Iranian teachers have been providing their students with online education during these days and the state television also broadcasted educational programs in which school books were taught. Referring to the significance of online teaching, the minister of education said that the officials have to ensure access to the Internet for all the Iranian students [41]. Based on the statistics provided by the Minister of Education on the 20th of April 2020, only 6.9% of students do not have access to the Internet [42]. This shows that near one-twentieth had accessed to our distributed questionnaire.

This project was limited in several ways. First, it was a cross-sectional study. The second and most important limitation lies in the fact that the Ministry of Education was not prepared enough for managing this crisis by prospective strategies at the time of our research survey. It was also not specifically designed to evaluate the students’ mental health characteristics; also, the present study was unable to investigate the participants’ financial status and did not include this variable in the results.

Because of the possible risks associated with the contraction of the disease, a community-based general sampling survey could not be performed; therefore, this cross-sectional web-based survey was utilized for data collection. The authors understood that in this method, the mentioned population would hold a selection bias and could not be a representative of the general population although it was the optimum approach during this critical period of time to limit the disease spread. Possible selection biases would be individuals with access to the Internet and among higher socioeconomic populations. However, based on a report on the 30th of March 2020, which during the final days of our survey, 93.1% of Iranian students had access to the Internet [43].

## Conclusion

This study has gone some way toward enhancing our understanding of the students’ significant behavioral and socializing changes during COVID-19 lockdown and represents the necessity of organizing critical strategies for managing children’s mental health. Switching factors like sleep pattern, screen time, and leisure seem to be the most significant variables, influencing the main consequences of school closure and lockdown period.

## Supplementary Information

**Additional file 1: Supplementary Figure 1.** Frequency of socializing during COVID-19 pandemic quarantine by students based on (A) Public or private school (B) Living location (C) Age group (D) Education level (E) Grade (F) Gender.

**Additional file 2: Supplementary Figure 2.** Frequency of activity preference among students during school closure based on gender

**Additional file 3: Supplementary Figure 3.** Frequency of the activity preference among students during school closure based on age (A) 6 to 9 years; (B) 10 to 12 years; (C) 13 to 15 years; (D) 16 and above years old

**Additional file 4: Supplementary Figure 4.** Frequency of activity preference among students during school closure based on age (A) 1 to 3; (B) 4 to 6; (C) 7 to 9; (D) and 10 to

**Additional file 5: Supplementary Figure 5.** Frequency of activity preference among students during school closure based on living location

**Additional file 6: Supplementary Figure 6.** Frequency of activity preference among students during school closure based on living location

**Additional file 7: Supplementary Figure 7.** Frequency of activity preference among students during school closure based on GPA: (A) Under 15; (B) 15 to 18; (C) Above 18

## Data Availability

Data are attached as supplementary materials, and information related to the study is in the manuscript. Please contact the corresponding author for any further data. Also, the questionnaire of this study has been added as supplementary data for further use in other studies.
