# Supplementary material for: Students’ attitude and sleep pattern during school closure following COVID-19 pandemic quarantine: a web-based survey in south of Iran
Source: Environ Health Prev Med. 2021 Mar 10;26:33. doi: 10.1186/s12199-021-00950-4 (PMC7945607; doi:10.1186/s12199-021-00950-4)
Supplement: Supplementary file 1 — Additional file 1: Supplementary Figure 1. Frequency of socializing during COVID-19 pandemic quarantine by students based on (A) Public or private school (B) Living location (C) Age group (D) Education level (E) Grade (F) Gender. [file 12199_2021_950_MOESM1_ESM.docx]

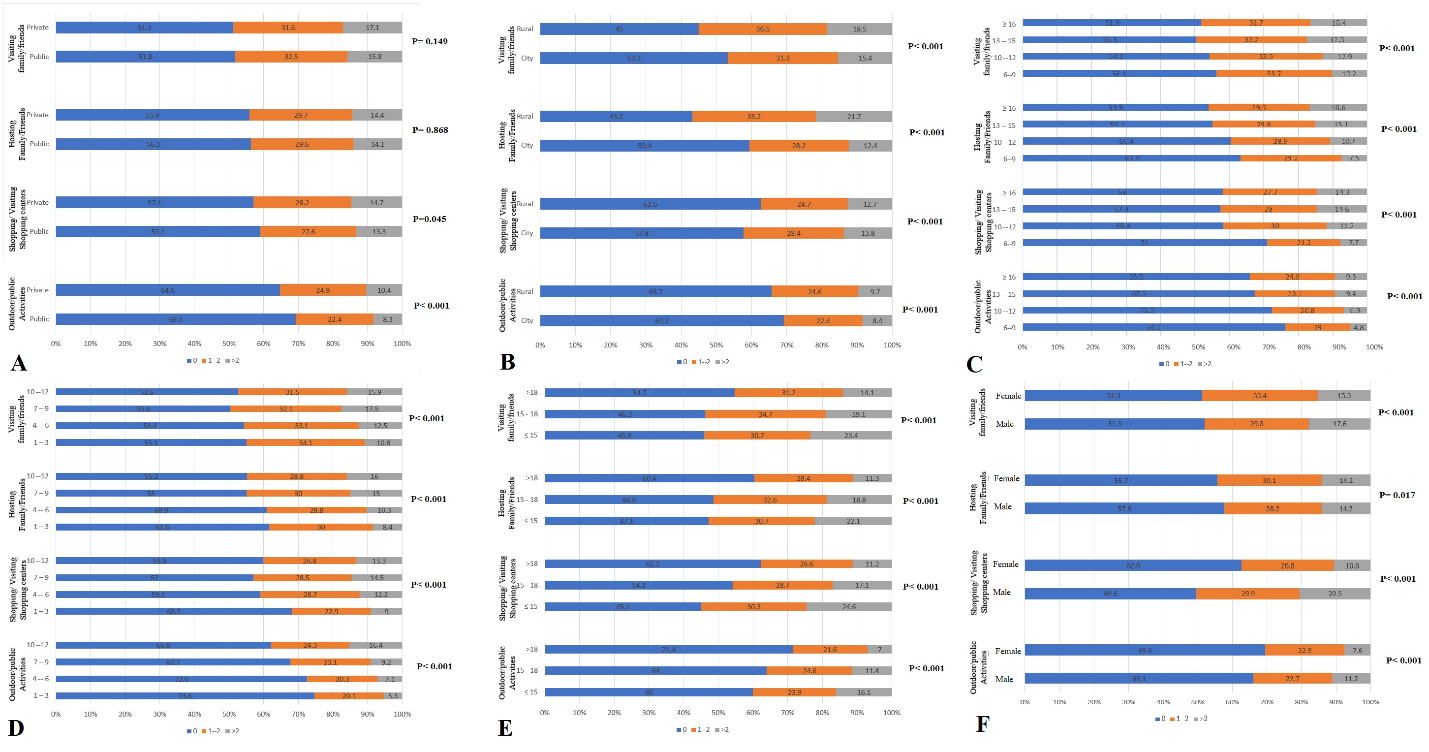


**Supplementary Figure 1.** Frequency of socializing during COVID-19 pandemic quarantine by students based on (A) Public or private school (B) Living location (C) Age group (D) Education level (E) Grade (F) Gender.
