# Supplementary material for: Students’ attitude and sleep pattern during school closure following COVID-19 pandemic quarantine: a web-based survey in south of Iran
Source: Environ Health Prev Med. 2021 Mar 10;26:33. doi: 10.1186/s12199-021-00950-4 (PMC7945607; doi:10.1186/s12199-021-00950-4)
Supplement: Supplementary file 2 — Additional file 2: Supplementary Figure 2. Frequency of activity preference among students during school closure based on gender [file 12199_2021_950_MOESM2_ESM.docx]

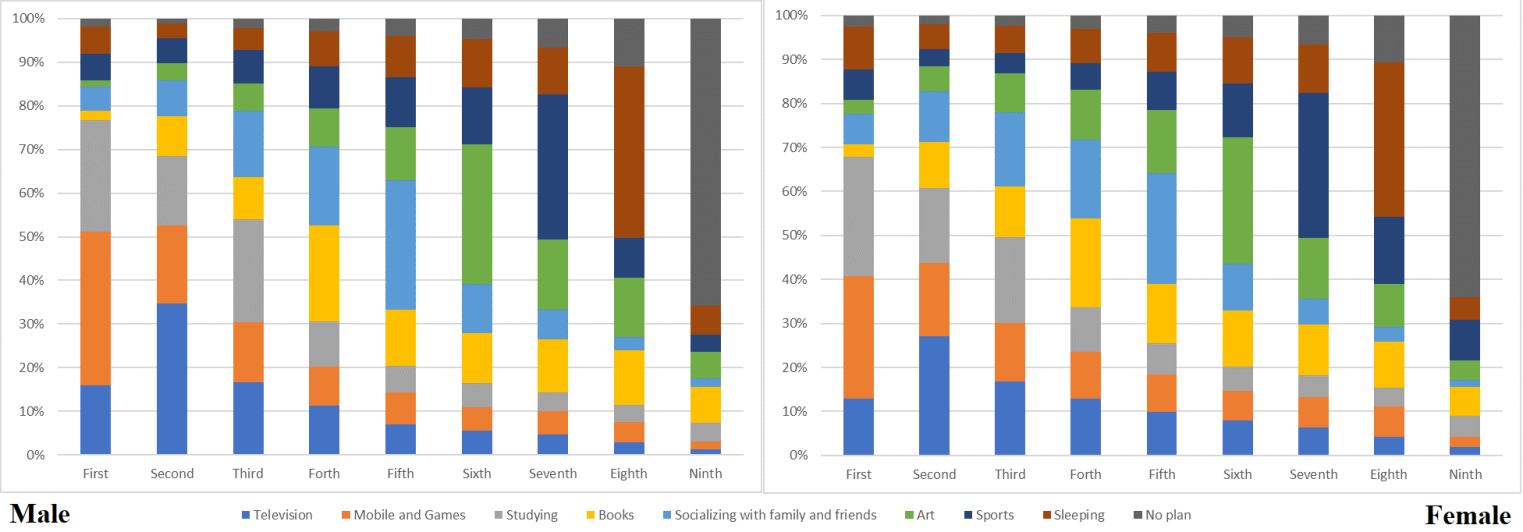


**Supplementary Figure 2.** Frequency of activity preference among students during school closure based on gender
