# Supplementary material for: Students’ attitude and sleep pattern during school closure following COVID-19 pandemic quarantine: a web-based survey in south of Iran
Source: Environ Health Prev Med. 2021 Mar 10;26:33. doi: 10.1186/s12199-021-00950-4 (PMC7945607; doi:10.1186/s12199-021-00950-4)
Supplement: Supplementary file 3 — Additional file 3: Supplementary Figure 3. Frequency of the activity preference among students during school closure based on age (A) 6 to 9 years; (B) 10 to 12 years; (C) 13 to 15 years; (D) 16 and above years old [file 12199_2021_950_MOESM3_ESM.docx]

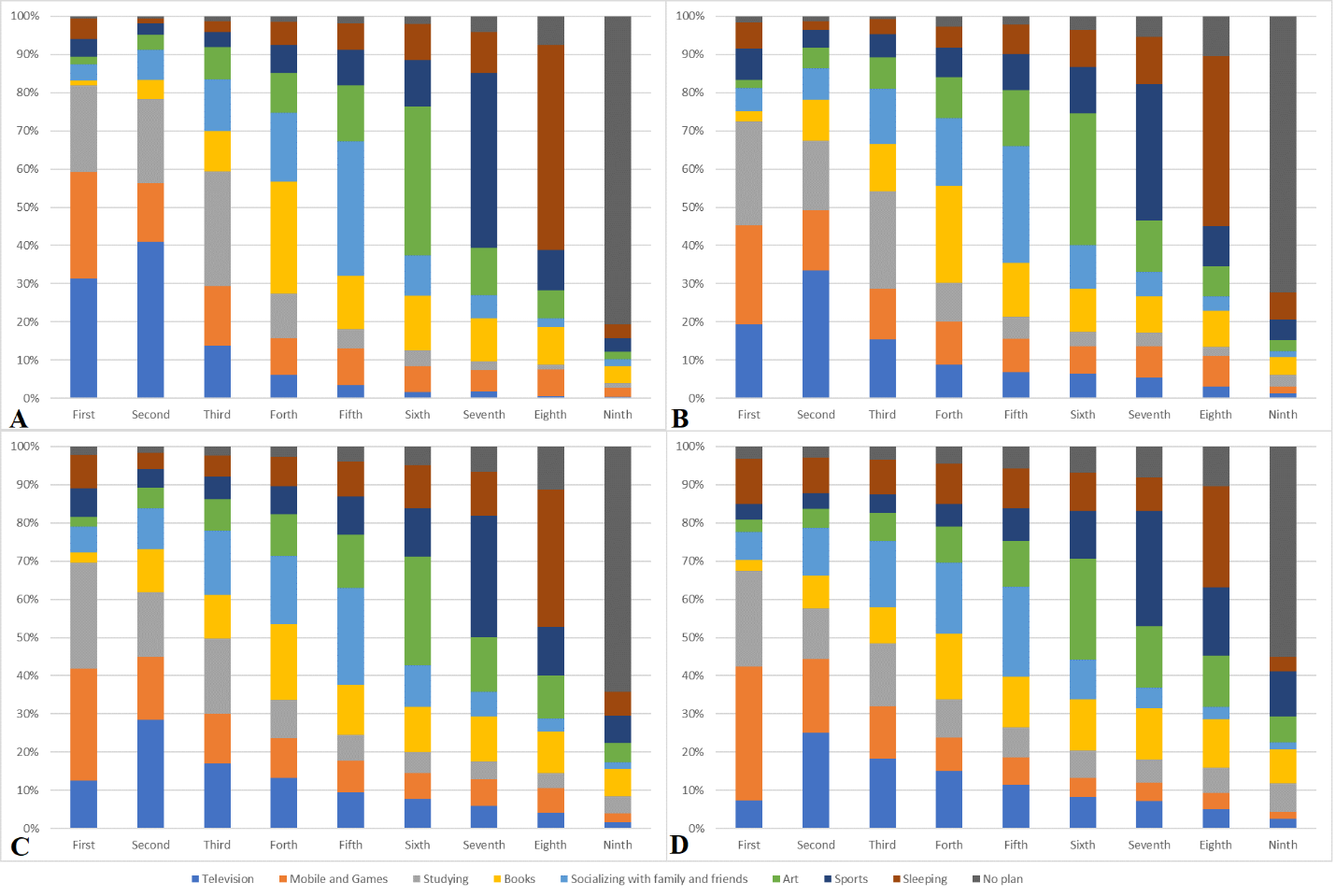


**Supplementary Figure 3.** Frequency of the activity preference among students during school closure based on age (A) 6 to 9 years; (B) 10 to 12 years; (C) 13 to 15 years; (D) 16 and above years old
