# Supplementary material for: Students’ attitude and sleep pattern during school closure following COVID-19 pandemic quarantine: a web-based survey in south of Iran
Source: Environ Health Prev Med. 2021 Mar 10;26:33. doi: 10.1186/s12199-021-00950-4 (PMC7945607; doi:10.1186/s12199-021-00950-4)
Supplement: Supplementary file 4 — Additional file 4: Supplementary Figure 4. Frequency of activity preference among students during school closure based on age (A) 1 to 3; (B) 4 to 6; (C) 7 to 9; (D) and 10 to [file 12199_2021_950_MOESM4_ESM.docx]

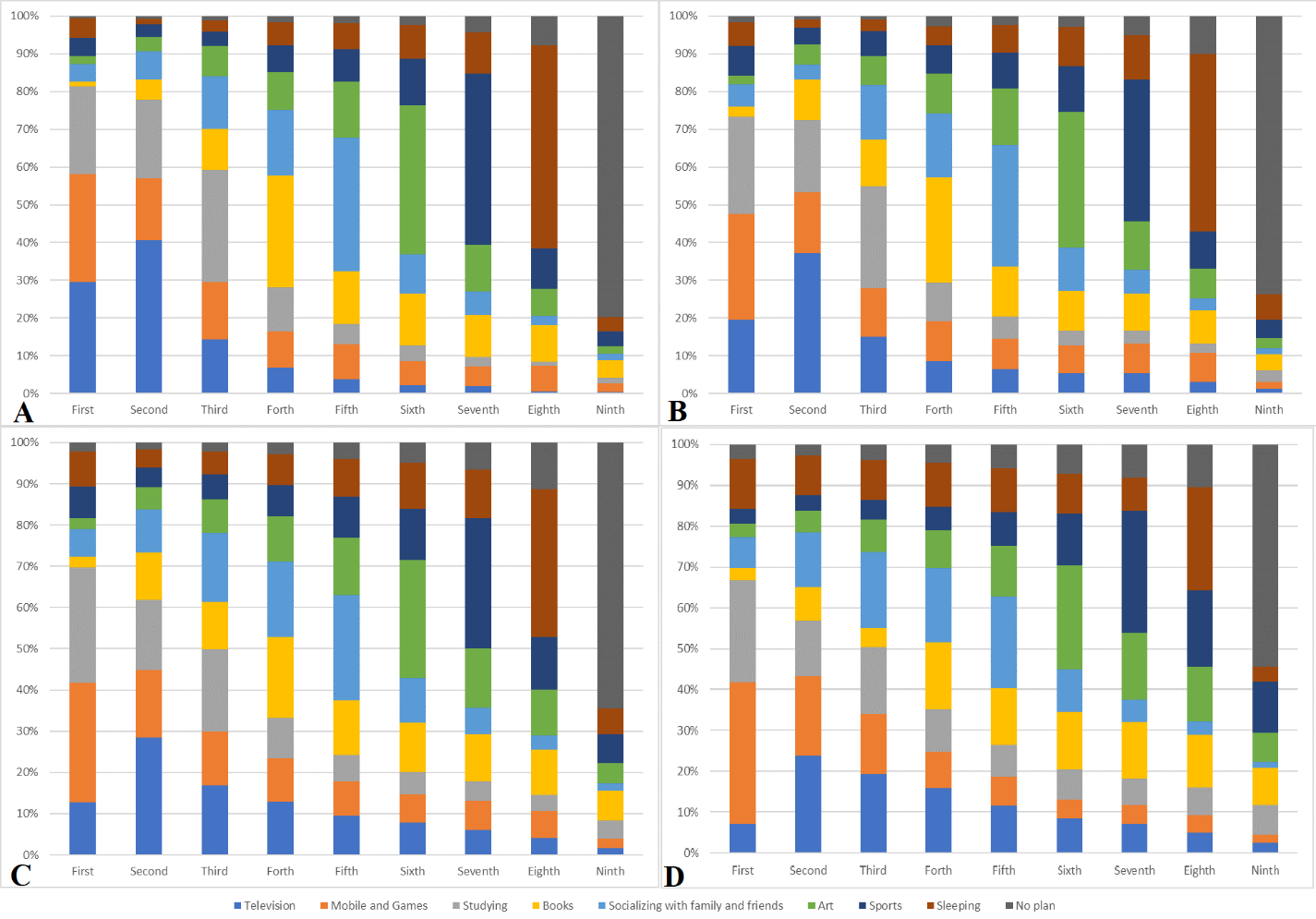


**Supplementary Figure 4.** Frequency of activity preference among students during school closure based on age (A) 1 to 3; (B) 4 to 6; (C) 7 to 9; (D) and 10 to 12 education level.
