# Supplementary material for: Students’ attitude and sleep pattern during school closure following COVID-19 pandemic quarantine: a web-based survey in south of Iran
Source: Environ Health Prev Med. 2021 Mar 10;26:33. doi: 10.1186/s12199-021-00950-4 (PMC7945607; doi:10.1186/s12199-021-00950-4)
Supplement: Supplementary file 7 — Additional file 7: Supplementary Figure 7. Frequency of activity preference among students during school closure based on GPA: (A) Under 15; (B) 15 to 18; (C) Above 18 [file 12199_2021_950_MOESM7_ESM.docx]

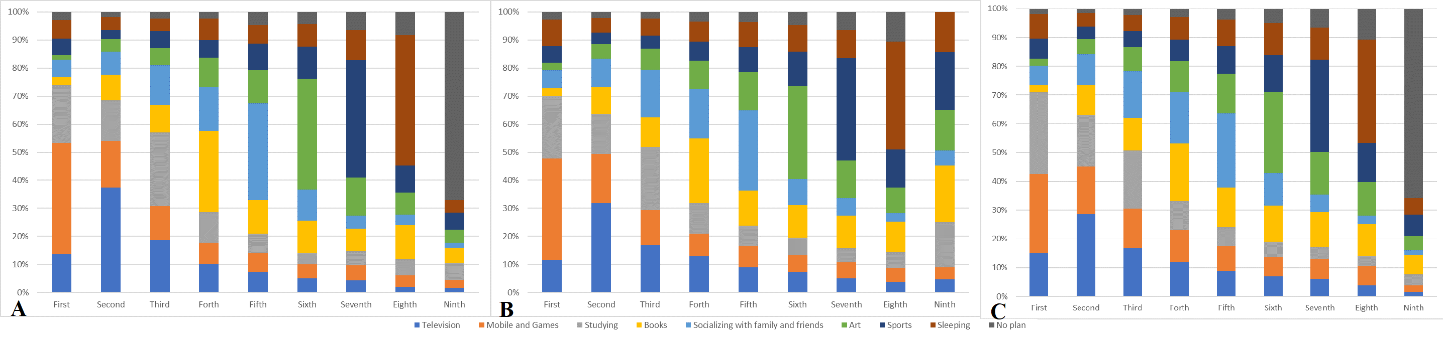


**Supplementary Figure 7.** Frequency of activity preference among students during school closure based on GPA: (A) Under 15; (B) 15 to 18; (C) Above 18
